# Supplementary material for: Single-cell analysis supports a luminal-neuroendocrine transdifferentiation in human prostate cancer
Source: Commun Biol. 2020 Dec 16;3:778. doi: 10.1038/s42003-020-01476-1 (PMC7745034; doi:10.1038/s42003-020-01476-1)
Supplement: Supplementary file 1 — Supplementary Information [file 42003_2020_1476_MOESM1_ESM.pdf]

Supplementary Information

Single-cell analysis supports a luminal-neuroendocrine trans-differentiation in human prostate cancer

Authors: Baijun Dong[2#], Juju Miao[1,3#], Yanqing Wang[2#], Wenqin Luo[1], Zhongzhong Ji[1], Huadong Lai[1,3], Man Zhang[1,3], Xiaomu Cheng[1,3], Jinming Wang[2], Yuxiang Fang[1,2], Helen He Zhu[1,2], Chee Wai Chua[1,2], Liancheng Fan[2], Yinjie Zhu[2], Jiahua Pan[2], Jia Wang[1,2]*, Wei Xue[2]*, Wei-Qiang Gao[1,3]*

Affiliations:

[1] State Key Laboratory of Oncogenes and Related Genes, Renji-Med-X Stem Cell Research Center, Department of Urology, Ren Ji Hospital, School of Medicine and School of Biomedical Engineering, Shanghai Jiao Tong University, Shanghai, 200127, China

[2] Department of Urology, Renji Hospital, School of Medicine, Shanghai Jiao Tong University, Shanghai 200127, China;

[3] School of Biomedical Engineering & Med-X Research Institute, Shanghai Jiao Tong University, Shanghai 200030, China;

[#]These authors contributed equally to this work.

***Correspondence author**: Jia Wang (wj860520@163.com), Wei Xue (xuewei@renji.com) or Wei-Qiang Gao (gao.weiqiang@sjtu.edu.cn).

Tel: 86-21-68383917, Fax: 86-21-68383916.

Address:

[1,3] Stem Cell Research Center, Ren Ji Hospital, 160 Pujian Rd., School of Medicine, Shanghai Jiao Tong University, Shanghai, 200127, China.

[2] Department Urology, Ren Ji Hospital, 160 Pujian Rd., School of Medicine, Shanghai Jiao Tong University, Shanghai, 200127, China.

Supplementary fig. 1

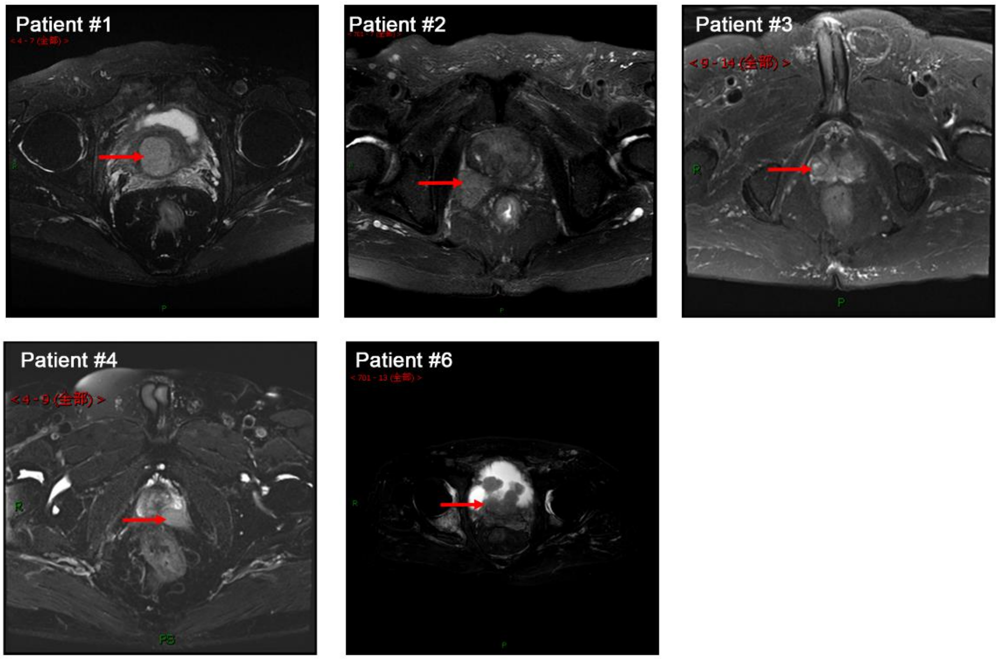

Fig.S1 CT images of 5 prostate tumors

CT images showing the tumor sites of five CRPC patients. Red arrows indicate the biopsy sites in each patient.

Supplementary fig. 2

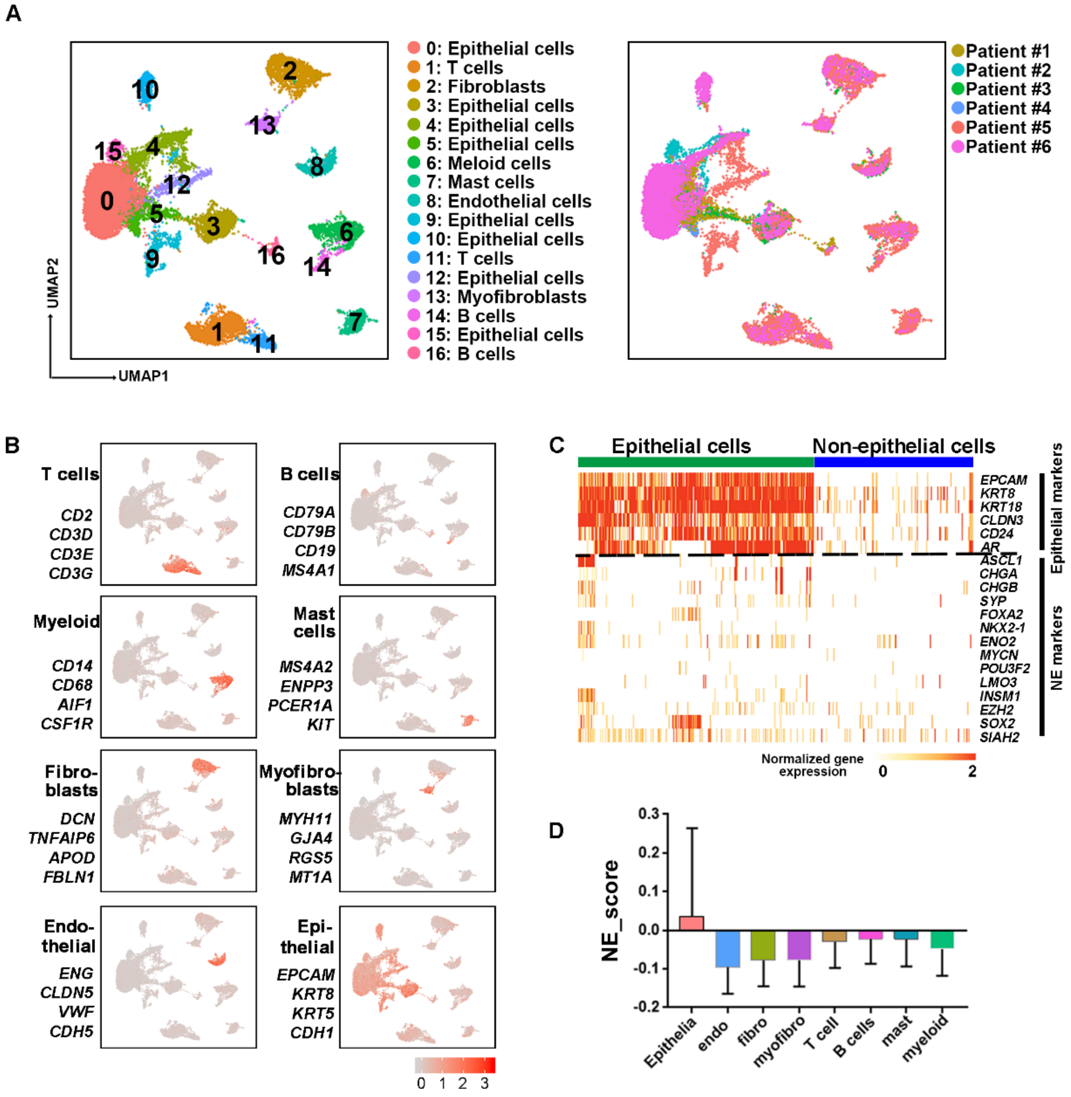

Fig.S2 Single-Cell Transcriptomic Profiling of 6 CRPC Tumors

(**A**) UMAP visualization of the 21,292 cells from 6 CRPC patients colored by clusters and sample origin, respectively. (**B**) UMAP plots of the 21,291 cells from 6 patients with cells colored by the score of marker gene sets for particular cell types (marker genes and associated cell types are indicated next to each plot). The minimum score is indicated by light grey and the maximum score is indicated by red. (**C**) Heatmap shows the expression level of epithelial lineage markers and NE markers. (**D**) Bar plot for NE index in cells that grouped by cell type (Data are mean ± SD, source data are provided as Supplementary Data 1).

Supplementary fig. 3

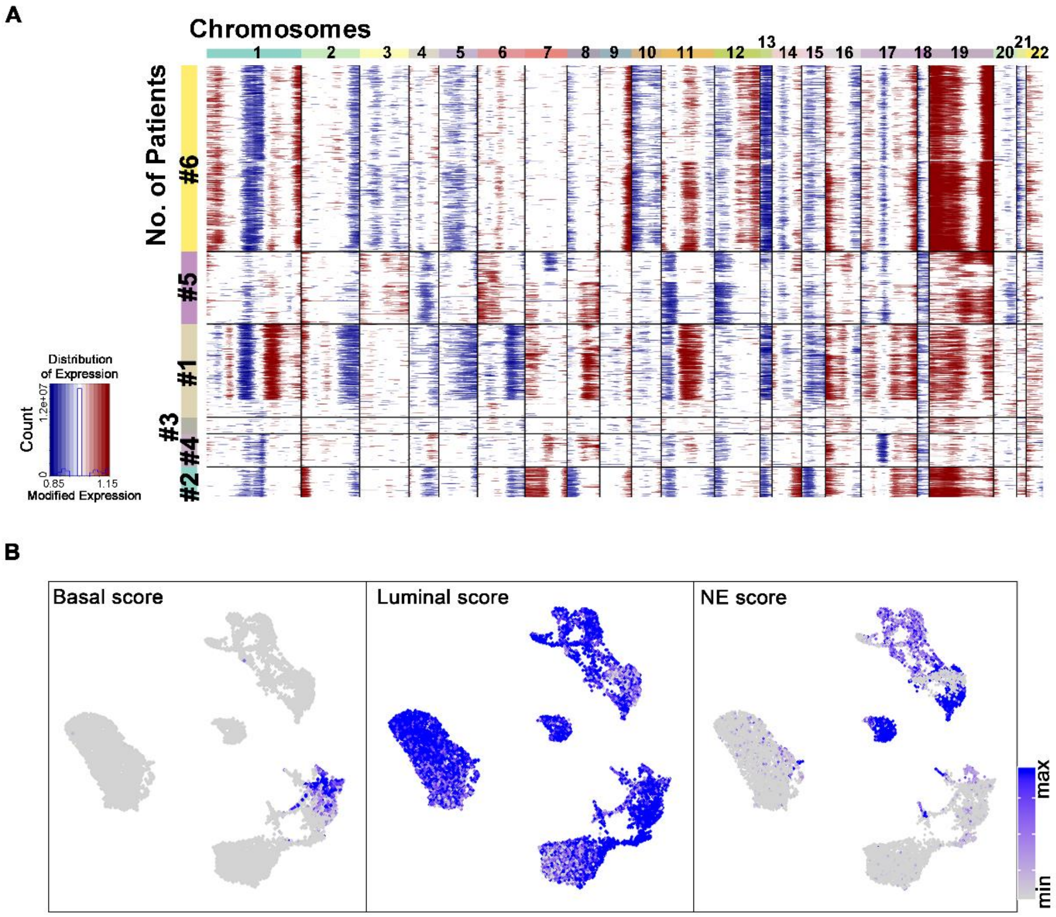

Fig.S3 Cell identity determination by inferred copy number variation (CNV) analysis

(**A**) Chromosomal landscape of inferred large-scale CNVs for tumor cells from 6 patients. The normal prostate epithelial cells from Henry dataset were set as "reference" cells [22], such that their average CNV value was subtracted from all epithelial cells from our dataset. The color bar in row indicates for 22 chromosomes while which in column indicates for corresponding patient. (**B**) UMAP visualization of all 12,861 epithelial cells for the 6 patients with cells colored by the gradient of basal score (left), luminal score (middle) and NE score (right).

Supplementary fig. 4

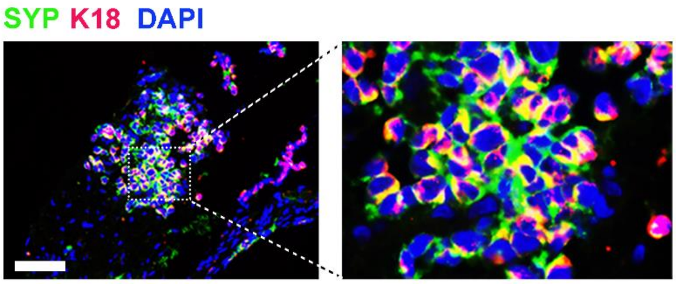

Fig.S4 Immunostaining of luminal marker K18 and NE marker SYP shows that the NE cell in patient #2 was luminal like phenotype.

Supplementary fig. 5

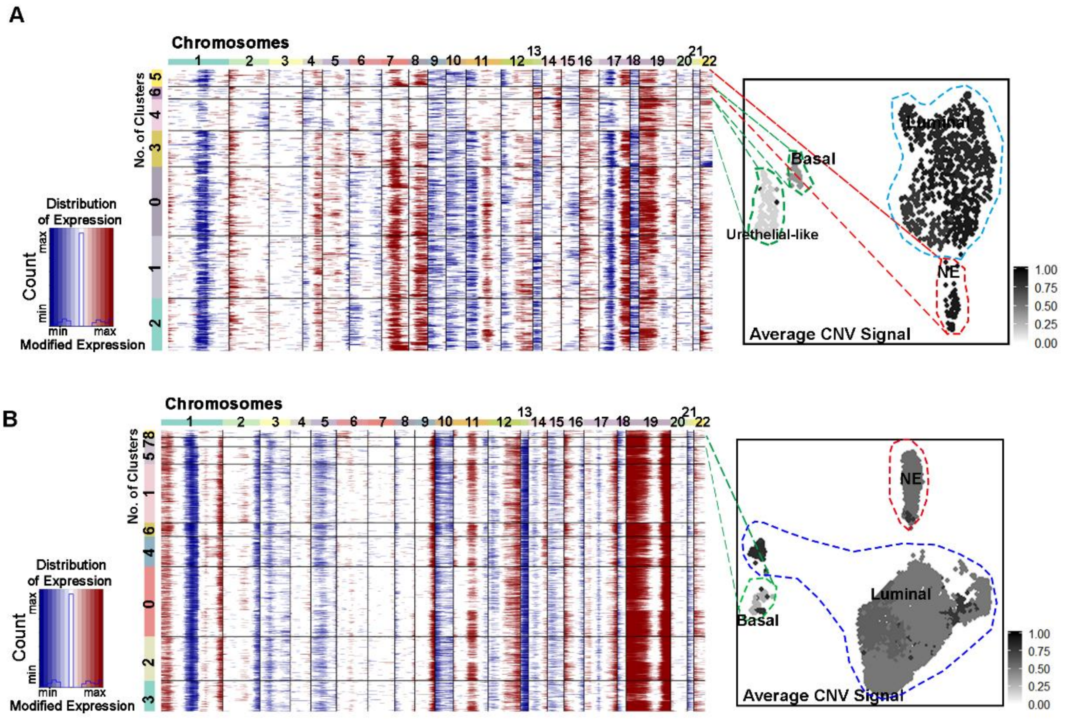

Fig.S5 Clonal analysis of patient #4 and #6 by inferred copy number variation (CNV) analysis

(**A** and **B**) Chromosomal landscape (left) of inferred large-scale CNVs for epithelial cells from patient #4 (A) and #6 (B), in which epithelial cells from 3 healthy men were set as the reference "normal" cells. The color bar in row indicates for 22 chromosomes while which in column indicates for corresponding Seurat cluster. UMAP visualization of average inferred CNV signals (right).

Supplementary Table 1: Information of 6 scRNA-Seq samples

Patient ID	Patient #1	Patient #2	Patient #3	Patient #4	Patient #5	Patient #6
Estimated Number of Cells by CellRanger	3961	1397	1517	1641	9821	8911
Mean Reads per Cell	86985	270,404	270127	241728	39732	52419
Median Genes per Cell	2595	4871	2595	3404	1457	2380
Valid Barcodes	97.40%	96.90%	95.70%	93.70%	96.00%	93.60%
Cells after QC filtering	3022	950	965	1062	8690	6603
Number/proportion of epithelial cells	2691/0.890	914/0.962	447/0.463	996/0.938	2123/0.244	5536/0.838
Number/proportion of fibroblasts	63/0.021	4/0.004	152/0.158	3/0.003	1340/0.154	306/0.046
Number/proportion of mast cells	0/0.000	0/0.000	0/0.000	0/0.000	1006/0.116	31/0.005
Number/proportion of T cells	50/0.017	2/0.002	17/0.018	3/0.003	2174/0.250	264/0.040
Number/proportion of endothelial cells	74/0.024	1/0.001	248/0.257	36/0.034	353/0.041	152/0.023
Number/proportion of myeloid cells	28/0.009	21/0.022	63/0.065	12/0.011	855/0.098	139/0.021
Number/proportion of myofibroblasts	18/0.006	0/0.000	21/0.022	5/0.005	316/0.036	157/0.024
Number/proportion of B cells	98/0.032	8/0.008	17/0.018	5/0.005	523/0.060	18/0.003

Supplementary Table 2: Marker genes used for scoring single cell

Basal	Luminal	Neuroendocrine	AR pathway	Stemness	EMT
KRT5	NKX3-1	ASCL1	AR	ALDH1A1	CDH2
KRT14	KRT8	CHGA	KLK3	CD44	CDH11
TP63	KRT18	CHGB	KLK2	PROM1	FN1
ITGA6	CD24	SYP	FKBP5	NANOG	VIM
KRT17	PSCA	FOXA2	TMPRSS2	KIT	TWIST1
KRT15	LMO7	NKX2-1	FOXA1	NES	SNAI1
DKK1	CLDN3	ENO2	GATA2	KLF4	ZEB1
CAV1	AMACR	MYCN	SLC45A3	CD55	ZEB2
CAV2	ERG	POU3F2		ALCAM	DCN
CYR61	ALDH1A3	NCAM1		NOTCH4	
		INSM1		WNT7A	
		EZH2		PDPN	
		SOX2			
		SIAH2			
