# Supplementary material for: Single-cell analysis supports a luminal-neuroendocrine transdifferentiation in human prostate cancer
Source: Commun Biol. 2020 Dec 16;3:778. doi: 10.1038/s42003-020-01476-1 (PMC7745034; doi:10.1038/s42003-020-01476-1)
Supplement: Supplementary file 2 — Description of Additional Supplementary Files [file 42003_2020_1476_MOESM2_ESM.pdf]

**Description of Additional Supplementary Files**

File Name: Supplementary Data 1
Description: Source data of the relevant figures.

File Name: Supplementary Data 2
Description: Metadata of scRNA-seq data from 6 CRPC samples.

File Name: Supplementary Data 3
Description: DEGs of each cluster in epithelial cells of 6 CRPC samples (Log2 fold change > 0.25, FDR < 0.001; one-tailed Wilcoxon rank sum test, P values adjusted for multiple testing using the Bonferroni correction).

File Name: Supplementary Data 4
Description: Characteristics of TMA from 297 patients.

File Name: Supplementary Data 5
Description: ScVelo_Gene_likelihood calculated by RNA velocity.

File Name: Supplementary Data 6
Description: This table contains the 30 genes with the highest NMF score for each factor in each sample. Association to meta-programs is indicated below.

File Name: Supplementary Data 7
Description: Pearson Correlation Coefficients of genes in P1, P2 and P4.
