# Supplementary material for: Single-cell analysis supports a luminal-neuroendocrine transdifferentiation in human prostate cancer
Source: Commun Biol. 2020 Dec 16;3:778. doi: 10.1038/s42003-020-01476-1 (PMC7745034; doi:10.1038/s42003-020-01476-1)
Supplement: Supplementary file 11 — Reporting Summary [file 42003_2020_1476_MOESM11_ESM.pdf]

nature research

Corresponding author(s): Wei-Qiang Gao

Last updated by author(s): Sep 20, 2020

Reporting Summary

Nature Research wishes to improve the reproducibility of the work that we publish. This form provides structure for consistency and transparency in reporting. For further information on Nature Research policies, see our Editorial Policies and the Editorial Policy Checklist.

Statistics

For all statistical analyses, confirm that the following items are present in the figure legend, table legend, main text, or Methods section.

n/a	Confirmed	
☐	☒	The exact sample size (*n*) for each experimental group/condition, given as a discrete number and unit of measurement
☐	☒	A statement on whether measurements were taken from distinct samples or whether the same sample was measured repeatedly
☐	☒	The statistical test(s) used AND whether they are one- or two-sided<br>*Only common tests should be described solely by name; describe more complex techniques in the Methods section.*
☒	☐	A description of all covariates tested
☐	☒	A description of any assumptions or corrections, such as tests of normality and adjustment for multiple comparisons
☐	☒	A full description of the statistical parameters including central tendency (e.g. means) or other basic estimates (e.g. regression coefficient) AND variation (e.g. standard deviation) or associated estimates of uncertainty (e.g. confidence intervals)
☐	☒	For null hypothesis testing, the test statistic (e.g. F, t, r) with confidence intervals, effect sizes, degrees of freedom and P value noted<br>*Give P values as exact values whenever suitable.*
☒	☐	For Bayesian analysis, information on the choice of priors and Markov chain Monte Carlo settings
☐	☒	For hierarchical and complex designs, identification of the appropriate level for tests and full reporting of outcomes
☐	☒	Estimates of effect sizes (e.g. Cohen's *d*, Pearson's *r*), indicating how they were calculated

Our web collection on statistics for biologists contains articles on many of the points above.

Software and code

Policy information about availability of computer code

Data collection	IF images were acquired using a Zeiss LSM 710 confocal microscope and were processed by ZEN Imaging Software. IHC images were acquired using an Olympus BX53 System Microscope.
Data analysis	Statistical analyses were performed using GraphPad Prism (v8.1.1) or R (v3.6.1) or Python (v3.7.6). Single-cell RNA sequencing data were analyzed by CellRanger (v2.1.1) and Seurat(v3.1.5), inferCNV(v1.4.0), scanpy(v1.4.5.1), nmf(v0.22.0), SCENIC(v1.1.2.2) and GSVA(v1.34.0). No custom code was used.

For manuscripts utilizing custom algorithms or software that are central to the research but not yet described in published literature, software must be made available to editors and reviewers. We strongly encourage code deposition in a community repository (e.g. GitHub). See the Nature Research guidelines for submitting code & software for further information.

Data

Policy information about availability of data

All manuscripts must include a data availability statement. This statement should provide the following information, where applicable:

- Accession codes, unique identifiers, or web links for publicly available datasets
- A list of figures that have associated raw data
- A description of any restrictions on data availability

single cell RNA-seq data have been deposited in NCBI Gene Expression Omnibus (GEO) database under accession number GSE137829.
Figures with associated raw data:
Figure1C
Figure2
Figure3A,B

April 2020

Figure4A,B,D,E
Figure5
Figure6A,B
Figure7
Supplementary figure2/3/5

Field-specific reporting

Please select the one below that is the best fit for your research. If you are not sure, read the appropriate sections before making your selection.

- [x] Life sciences
- [ ] Behavioural & social sciences
- [ ] Ecological, evolutionary & environmental sciences

For a reference copy of the document with all sections, see nature.com/documents/nr-reporting-summary-flat.pdf

Life sciences study design

All studies must disclose on these points even when the disclosure is negative.

Sample size	Sample size for 10x Genomics scRNA-seq and PC TMAs were determined by the availability of patient samples. Statistical methods were not used to determine 10X sample size but it was sufficient for this proof-of-concept study.<br>The exact number of samples used for single-cell RNA sequencing is 6 CRPC biopsy tissues and 297 prostate cancer resection tissues
Data exclusions	CellRanger filters any barcode that contains less than 10% of the 99th percentile of total UMI counts per barcode, as these are considered to be associated with low quality cell barcodes. In Seurat-based filtering, we filter cells based on the number of detected genes per cell (500 to 7000) and the percentage of mitochondrial genes expressed (<10%). The mitochondrial genes and ribosomal genes were also removed from the gene expression matrix.
Replication	No replication was performed due to the sample limitation.
Randomization	This is not relevant to our study due to the available of patients.
Blinding	Not applicable since there was no specific grouping.

Reporting for specific materials, systems and methods

We require information from authors about some types of materials, experimental systems and methods used in many studies. Here, indicate whether each material, system or method listed is relevant to your study. If you are not sure if a list item applies to your research, read the appropriate section before selecting a response.

Materials & experimental systems

n/a	Involved in the study	
[ ]	[x]	Antibodies
[x]	[ ]	Eukaryotic cell lines
[x]	[ ]	Palaeontology and archaeology
[x]	[ ]	Animals and other organisms
[ ]	[x]	Human research participants
[x]	[ ]	Clinical data
[x]	[ ]	Dual use research of concern

Methods

n/a	Involved in the study	
[x]	[ ]	ChIP-seq
[x]	[ ]	Flow cytometry
[x]	[ ]	MRI-based neuroimaging

Antibodies

Antibodies used	anti-SOX2 (Abcam,ab92494),<br>anti-AR (Abcam, EPR1535(2)),<br>anti-CK5 (Biolegend, 905904),<br>anti-SYP (Cell Signaling Technology,#36406),<br>anti-K18 (ProteinTech, 66187-1-Ig).
Validation	anti-SOX2 (Abcam,ab92494), validation references Pubmed ID:30213750,30559277;<br>anti-AR (Abcam, EPR1535(2)), validation references Pubmed ID:30580238,30976175;<br>anti-CK5 (Biolegend, 905904), validation reference Pubmed ID:30304685;<br>anti-SYP (Cell Signaling Technology,#36406), validation reference Pubmed ID:32286280;<br>anti-K18 (ProteinTech, 66187-1-Ig), validation reference Pubmed ID: 31886853

Human research participants

Policy information about studies involving human research participants

Population characteristics	Six male patients with prostate cancer diagnosis were enrolled in this study. Detailed information could be found in table1 of manuscript.
Recruitment	All patients were recruited and managed by Renji hospital,the participating male patients were required to meet the following requirements: 1) the patients must have developed resistance to castration therapy; 2) CT imaging showed an apparent prostate tumor. In addition, we preferentially selected patients whose circulating PSA level was lower than 20 ng/ml.
Ethics oversight	The present study was approved by the Institutional Ethics Review Board of Ren Ji Hospital, Shanghai Jiao Tong University School of Medicine, and written informed consent was obtained from every patient.

Note that full information on the approval of the study protocol must also be provided in the manuscript.
